# MarkerMap: nonlinear marker selection for single-cell studies

**DOI:** 10.1038/s41540-024-00339-3

**Published:** 2024-02-14

**Authors:** Wilson Gregory, Nabeel Sarwar, George Kevrekidis, Soledad Villar, Bianca Dumitrascu

**Affiliations:** 1 Department of Applied Mathematics and Statistics, Johns Hopkins University, Baltimore, MD 21218 USA; 2 Center for Data Science, New York University, New York, NY 10012 USA; 3 Mathematical Institute for Data Science, Johns Hopkins University, Baltimore, MD 21218 USA; 4 Department of Statistics, Columbia University, New York, NY 10027 USA; 5 Irving Institute for Cancer Dynamics, Columbia University, New York, NY 10027 USA

**Keywords:** Software, Computer science

## Abstract

Single-cell RNA-seq data allow the quantification of cell type differences across a growing set of biological contexts. However, pinpointing a small subset of genomic features explaining this variability can be ill-defined and computationally intractable. Here we introduce MarkerMap, a generative model for selecting minimal gene sets which are maximally informative of cell type origin and enable whole transcriptome reconstruction. MarkerMap provides a scalable framework for both supervised marker selection, aimed at identifying specific cell type populations, and unsupervised marker selection, aimed at gene expression imputation and reconstruction. We benchmark MarkerMap’s competitive performance against previously published approaches on real single cell gene expression data sets. MarkerMap is available as a pip installable package, as a community resource aimed at developing explainable machine learning techniques for enhancing interpretability in single-cell studies.

## Introduction

Recent advances in genomics and microscopy enable the collection of single cell gene expression data (scRNA-seq) across cells from spatial^1^ and temporal^2^ coordinates. Understanding how cells aggregate information across spatio-temporal scales and how, in turn, gene expression variability reflects this aggregation process remains challenging. A particular experimental design challenge is due to the fact that existing techniques (e.g., smFish^3^, seqFish^4^, MERFISH^5^, ISS^6^) rely on the pre-selection of a small number of target genes or *markers*, incapable of capturing the full transcriptomic information required to characterize subtle differences in cell populations. Selecting the best such markers (*marker selection*) is often statistically and computationally challenging, often a function of the nonlinearity of the data and the type of differences to be captured.

Marker selection is the product of both prior knowledge and computational analysis of previously collected scRNA-seq data. Computationally, it aims to reduce the dimension of data such as gene expression—from thousands of genes to a few—to enable downstream analysis such as visualization, cell type recovery, identification of gene programs or gene panel design for interventional studies. Akin to principal component analysis (PCA)^7^ or variational autoencoders (VAE)^8^, both popular in the analysis of single-cell RNA-seq^9,10^, marker selection methods seek to describe cells as datapoints in a space of few coordinates. To this end, PCA and VAE based methodologies associate cells with a smaller set of latent coordinates representing aggregates of weighted groups of gene expression. In contrast, marker selection approaches seek interpretable representations, where coordinates represent genes directly, rather than linear or nonlinear combinations of genes.

Many methods have been proposed to select markers that best differentiate between a set of discrete, pre-defined cell type classes^11–16^. These fall into two broad categories—one-vs-all and gene panel methods. One-vs-all methods are most common^11–13^ and seek to determine, for each cell type, a set of genes that are differentially expressed in that *one* cell type alone, when compared with *all* the other cell types. In particular, RankCorr^15^, a sparse selection approach inspired by the success of a related proteomic application^17^, offers theoretical guarantees and excellent experimental performance. Another recent algorithm with good performance, SMaSH^16^, uses a neural network framework leveraging techniques from the explainable machine learning literature^18^. In contrast, gene panel methods seek to identify groups of genetic markers that jointly distinguish across cell types. ScGeneFit^14^, for instance, is a compressive classification method^19^ which employs linear programming to select markers that preserve the classification structure of the data, without identifying genes with individual cell types, and possibly selecting fewer genes as a result. One-vs-all and gene panel alike, these methods are supervised: they rely on a ground truth classification structure of the cells. Few unsupervised techniques exist—SCMER^20^ is, to the best of our knowledge, the only genetic marker selection approach proposed that avoids explicit clustering by using nonlinear dimensionality reduction (UMAP) and manifold learning. Recent reviews on feature selection in genomics applications^21,22^ compare and contrast these marker selection methodologies in supervised, linear contexts.

More broadly, diverse solutions have been proposed to address the feature selection problem in non-genomic contexts. In linear settings, these include the popular *ℓ*_1_ regularization or Lasso^23^, and CUR decomposition^24^, while in nonlinear regression settings, outcomes are often predicted with neural networks^25^. In language models, explainable deep learning algorithms have been developed to predict and explain outcomes like review ratings or interview outcomes from texts where few significant words get highlighted as explanations for the outcome^26–29^. In imaging, given a trained model one can use shapley coefficients to identify parts of an image that produce a certain prediction^30,31^.

In this paper, we introduce MarkerMap, a scalable and generative framework for nonlinear marker selection. Our objectives are two-fold: (a) to provide a general method allowing joint marker selection and full transcriptome reconstruction, and (b) to compare and contrast tools across different communities—computational biology and explainable machine learning—within a single, accessible computational framework centered around transcriptomic studies. As a result, MarkerMap exhibits several key features. First, MarkerMap scales to large data sets without the need for ad-hoc gene pruning. Second, it provides a joint setting for both supervised and unsupervised learning. Third, it is generative, allowing for imputation to whole transcriptome levels from a reduced, informative number of markers. We provide a set of metrics to evaluate the quality of the imputations and compare the distributions of original transcriptomes with their reconstructions. Forth, its supervised option robustly tolerates small rates of labeling misclassification, which could emerge from processing and cell type assignment errors. We apply MarkerMap to real data, including cord blood mononuclear cells (CBMCs) assayed with different technologies, longitudinal samples from mouse embryogenesis, and a developmental mouse brain single cell gene expression resource. Finally, a strong link exists between marker selection and the wider explainable machine learning literature^27,28^. As both communities are rapidly evolving, there is an increasing need to systematically compare new and existing methods, with the goal of understanding their strengths and limitations. To address this need, we benchmark MarkerMap against existing marker selection approaches and related methodologies from the wider explainable machine learning literature. We make MakerMap available as a pip installable package.

## Results

### MarkerMap: learning relevant markers for scRNA-seq studies

We developed MarkerMap, a generative, deep learning marker selection framework which uses scRNA-seq data to extract a small number of genes which non-linearly combine to allow whole transcriptome reconstruction, without sacrificing accuracy on downstream prediction tasks. The input to MarkerMap is log normalized scRNA-seq data, a budget $k\in {\mathbb{N}}$, and an optional annotation for each cell. MarkerMap then outputs a set of *k* genes (markers) which are most predictive of the output, together with the option of a non-linear map for reconstructing the original gene expression space.

Intuitively, MarkerMap computes feature importance scores for each gene in the input data using neural networks. These importance scores or logits (*Methods*) inform which genes are selected as representative of the input signal. MarkerMap then uses this reduced representation to compute an objective function predicting the given cell annotations (supervised; *Methods*), reconstructing the full input signal (unsupervised; *Methods*), or both (mixed strategy; *Methods*). The selection step is probabilistic and is achieved through sampling from a discrete distribution which allows end-to-end optimization over the selection and predictive steps. The learnt mappings allow (a) extracting the features most informative of a given annotation and (b) generating full gene expression profiles when information from only the marker set is available.

Technically, MarkerMap is an interpretable dimensionality reduction method based on the statistical framework of differentiable sampling optimization^26,28^. Targeted at addressing explainability tasks in machine learning, such methods have primarily been developed with text data in mind. Their performance has hence not been previously evaluated in a comprehensive way in the context of single cell studies. The relationship of MarkerMap with respect to these method and other previous approaches is discussed in *Methods* and Tables 1, 2, and 3.

**Table 1:** Classification performance metrics

Models	CITE-seq	Mouse Brain	Mouse Brain (subtypes)	Paul	Zeisel	Zeisel (subtypes)	SSv4
MarkerMap unsup	(0.831,0.809)	(0.982,0.981)	(0.812,0.805)	(0.660,0.639)	(0.793,0.782)	(0.437,0.392)	(0.694,0.680)
MarkerMap sup	(**0.939,0.931**)	(**0.994,0.994**)	(**0.863,0.856**)	(0.749,0.738)	(0.950,0.950)	(**0.686,0.664**)	(**0.857,0.853**)
MarkerMap joint	(0.859,0.838)	(0.983,0.983)	(0.811,0.804)	(0.623,0.598)	(0.771,0.759)	(0.466,0.420)	(0.767,0.758)
Random Markers	(0.822,0.798)	(0.760,0.735)	(0.387,0.359)	(0.537,0.510)	(0.734,0.715)	(0.401,0.347)	(0.287,0.256)
LassoNet	(0.937,0.927)	(0.984,0.984)	(0.839,0.832)	(**0.776,0.768**)	(0.944,0.942)	(0.676,0.653)	(0.797,0.790)
Concrete VAE	(0.811,0.784)	(0.786,0.765)	(0.403,0.376)	(0.533,0.503)	(0.726,0.710)	(0.396,0.342)	(0.309,0.277)
Global-Gumbel VAE	(0.812,0.785)	(0.785,0.763)	(0.406,0.376)	(0.557,0.527)	(0.724,0.707)	(0.384,0.327)	(0.304,0.267)
SMaSH	(0.930,0.918)	(0.976,0.975)	(0.845,0.835)	(0.755,0.734)	(0.952,0.952)	(0.674,0.655)	(0.833,0.827)
RankCorr	(0.866,0.856)	(0.927,0.926)	(0.622,0.608)	(0.673,0.660)	(0.946,0.946)	(0.588,0.557)	(0.616,0.592)
Scanpy *t*-test	(0.921,0.906)	(0.982,0.982)	(0.837,0.826)	(0.746,0.720)	(**0.960,0.960**)	(0.600,0.576)	(0.794,0.785)
Scanpy overestim_var	(0.920,0.904)	(0.975,0.975)	(0.814,0.805)	(0.749,0.727)	(0.953,0.953)	(0.630,0.611)	(0.747,0.735)
Scanpy Wilcoxon	(0.918,0.903)	(0.978,0.977)	(0.822,0.811)	(0.754,0.730)	(0.956,0.956)	(0.647,0.626)	(0.758,0.744)
Scanpy Wilcoxon Tie	(0.918,0.904)	(0.962,0.961)	(0.611,0.610)	(0.760,0.744)	(0.951,0.951)	(0.610,0.591)	(0.594,0.580)
COSG	(0.904,0.890)	(0.953,0.953)	(0.559,0.568)	(0.731,0.721)	(0.948,0.948)	(0.593,0.566)	(0.569,0.551)
PERSIST unsup	(0.869,0.850)	(0.977,0.976)	(0.800,0.792)	(0.657,0.624)	(0.873,0.873)	(0.484,0.448)	(0.718,0.703)
PERSIST sup	(0.912,0.896)	(0.987,0.987)	(0.836,0.828)	(0.685,0.662)	(0.922,0.922)	(0.562,0.533)	(0.788,0.778)

Average accuracy (first) and weighted F1 (second) scores across real single cell RNA-seq data sets, using a nearest neighbor classifier. All methods are instructed to select 50 markers. Higher values are better, and the top performer for each data set is bolded. Results are averaged over 10 runs.

**Table 2:** Full transcriptome reconstruction

Models	CITE-seq	Mouse Brain	Mouse Brain (subtypes)	Paul	Zeisel	Zeisel (subtypes)	SSv4
MarkerMap unsup	(0.711,0.656)	(**0.854,0.687**)	(**0.890,0.643**)	(0.912,0.577)	(0.609,**0.610**)	(0.615,0.613)	(**0.854,0.721**)
Random Markers	(**0.707,0.653**)	(0.885,0.706)	(0.928,0.665)	(0.925,0.584)	(0.613,0.615)	(0.616,0.615)	(0.909,0.754)
Scanpy HVGs	(0.718,0.665)	(0.895,0.723)	(0.927,0.673)	(0.926,0.592)	(0.636,0.632)	(0.635,0.630)	(0.877,0.740)
PERSIST unsup	(0.716,0.657)	(0.860,0.693)	(0.898,0.650)	(**0.903,0.571**)	(**0.604**,0.612)	(**0.607,0.612**)	(0.869,0.732)

Average *ℓ*_2_ (first) and *ℓ*_1_ (second) loss of single cell RNA-seq data sets to ones reconstructed from selected markers using a linear regression model. All methods are instructed to select 50 markers. Lower values are better, and the top performer for each data set is bolded. Results are averaged over 10 runs.

**Table 3:** Random Forest classification performance metrics

Models	CITE-seq	Mouse Brain	Mouse Brain (subtypes)	Paul	Zeisel	Zeisel (subtypes)	SSv4
MarkerMap unsup	(0.888,0.857)	(0.983,0.982)	(0.843,0.835)	(0.814,0.803)	(0.866,0.856)	(0.503,0.420)	(0.795,0.780)
MarkerMap sup	(**0.939,0.925**)	(**0.994,0.994**)	(**0.884,0.878**)	(0.882,0.879)	(0.945,0.944)	(0.717,0.683)	(**0.874,0.867**)
MarkerMap joint	(0.892,0.863)	(0.987,0.986)	(0.842,0.835)	(0.737,0.712)	(0.813,0.799)	(0.481,0.398)	(0.799,0.785)
Random Markers	(0.873,0.836)	(0.853,0.844)	(0.494,0.451)	(0.589,0.546)	(0.803,0.790)	(0.478,0.387)	(0.440,0.376)
LassoNet	(0.938,0.923)	(0.987,0.986)	(0.863,0.856)	(0.887,0.884)	(0.946,0.944)	(0.707,0.676)	(0.834,0.824)
Concrete VAE	(0.873,0.837)	(0.866,0.857)	(0.511,0.471)	(0.620,0.576)	(0.812,0.797)	(0.483,0.398)	(0.436,0.373)
Global-Gumbel VAE	(0.872,0.837)	(0.852,0.842)	(0.489,0.442)	(0.597,0.549)	(0.785,0.768)	(0.468,0.381)	(0.496,0.440)
SMaSH	(0.936,0.920)	(0.982,0.982)	(0.875,0.868)	(0.881,0.878)	(0.952,0.951)	(**0.722,0.693**)	(0.861,0.852)
RankCorr	(0.886,0.860)	(0.941,0.940)	(0.724,0.709)	(0.787,0.777)	(0.944,0.943)	(0.615,0.557)	(0.698,0.669)
Scanpy *t*-test	(0.930,0.911)	(0.988,0.988)	(0.870,0.862)	(0.892,0.889)	(**0.956,0.955**)	(0.687,0.655)	(0.834,0.824)
Scanpy overestim_var	(0.929,0.909)	(0.981,0.981)	(0.858,0.851)	(**0.894,0.891**)	(0.954,0.953)	(0.699,0.671)	(0.793,0.777)
Scanpy Wilcoxon	(0.926,0.905)	(0.983,0.982)	(0.864,0.857)	(0.891,0.887)	(0.951,0.951)	(0.713,0.683)	(0.807,0.793)
Scanpy Wilcoxon Tie	(0.924,0.902)	(0.971,0.971)	(0.621,0.620)	(0.891,0.889)	(0.951,0.950)	(0.689,0.661)	(0.612,0.601)
COSG	(0.919,0.894)	(0.962,0.962)	(0.570,0.572)	(0.853,0.852)	(0.950,0.949)	(0.665,0.627)	(0.592,0.573)
PERSIST unsup	(0.901,0.875)	(0.975,0.974)	(0.825,0.816)	(0.725,0.703)	(0.893,0.889)	(0.573,0.517)	(0.779,0.760)
PERSIST sup	(0.924,0.905)	(0.987,0.987)	(0.861,0.854)	(0.744,0.731)	(0.927,0.925)	(0.618,0.569)	(0.829,0.816)

Average accuracy (first) and weighted F1 (second) scores across real single cell RNA-seq data sets, using a Random Forest classifier. All methods are instructed to select 50 markers. Higher values are better, and the top performer for each data set is bolded. Results are averaged over 10 runs.

**Fig. 1:**
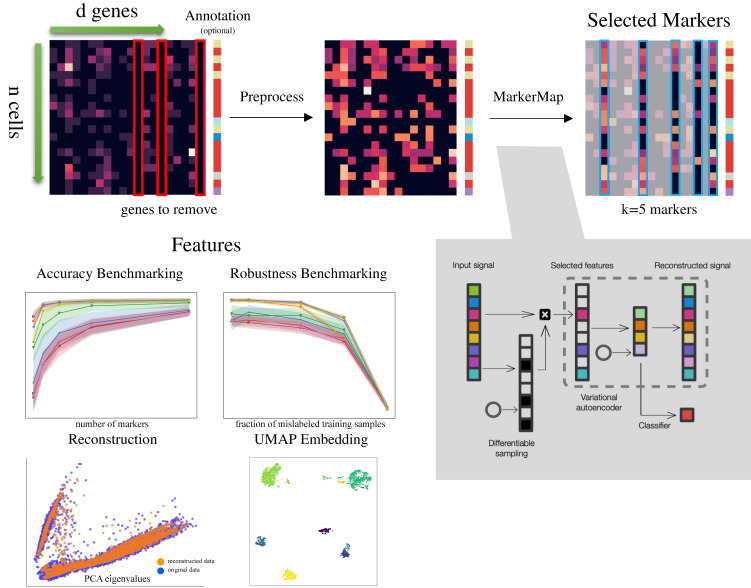
Computational pipeline of MarkerMap. Data are imported as an *n* × *d* array of expression counts, together with optional annotations. During preprocessing, some genes are removed, and the rest undergo scaling, normalization, and a log(1+X) transform (*Methods*). Then MarkerMap or a variety of other marker selection algorithms are run to pick *k* markers. These markers are used for downstream tasks including benchmarking, UMAP embedding, and data reconstruction. The architecture of MarkerMap is depicted in the lower right. Given input signals, a differentiable sampling process selects a global set of markers. In the supervised setting when annotations are available, the signal restricted to the selected markers is fed to a neural network that predicts labels. In the unsupervised version, the signal restricted to the selection is fed to a variational autoencoder that aims to reconstruct the original signal with no information of the label. The joint loss version uses a convex combination of the reconstruction loss and the classification loss. A circle represents a source of random inputs to be used for differentiable sampling, a technique for iteratively assigning weight to informative features (*Methods*).

MarkerMap is available as a well documented open-source software, along with tutorial and example workflows. The package provides a framework for custom designed feature selection methods along with metrics for evaluation (Fig. 1).

### Improving accuracy in supervised scRNA-seq studies

We evaluated the performance of MarkerMap in the context of five publicly available scRNA-seq studies: Zeisel^32^, a CITE-seq technology based data set^33^, a mouse brain scRNA-seq data set^34^, the Paul15 stem cell data set^35^, and the SSv4 V1 data set^36^ (see *Methods* for a full description of the data sets and the data processing pipeline).

MarkerMap’s performance is benchmarked against other non-linear approaches which, despite addressing related tasks, have not been previously compared to one another. In detail, we considered the following feature selection baselines (*Methods*): PERSIST^37^, LassoNet^25^, SMaSH^16^, and Concrete VAE^28^. We also adapted a continuous relaxation Gumbel-Softmax technique from^27^ to allow for global feature selection, rather than local selection, in an effort to quantify the effect of the different sampling techniques on downstream clustering performance; we refer to this method as Global-Gate or Global-Gumbel VAE. Finally, we quantified the quality of the learnt markers against those learnt through classical, non-generative methods – differential expression methods like COSG^38^ and the *t*-test, *t*-test with overestimated variances, Wilcoxon-ranked sum test, as well as a Wilcoxon-ranked sum test with tie correction from Scanpy^39^.

We report average misclassification and average F1 scores corresponding to a random forest classifier (Table 1) and a nearest neighbor classifier (Table 2), across single cell data sets. We find that MarkerMap performs competitively with respect to these metrics, often improving on state of the art techniques. It is worth noting that, similar to empirical studies where dimensionality reduction is shown to improve the accuracy of downstream classification tasks^40^, the accuracy of the classifier trained only on features detected by MarkerMap is often as good, or better, than that of the classifier trained on the full input.

**Fig. 2:**
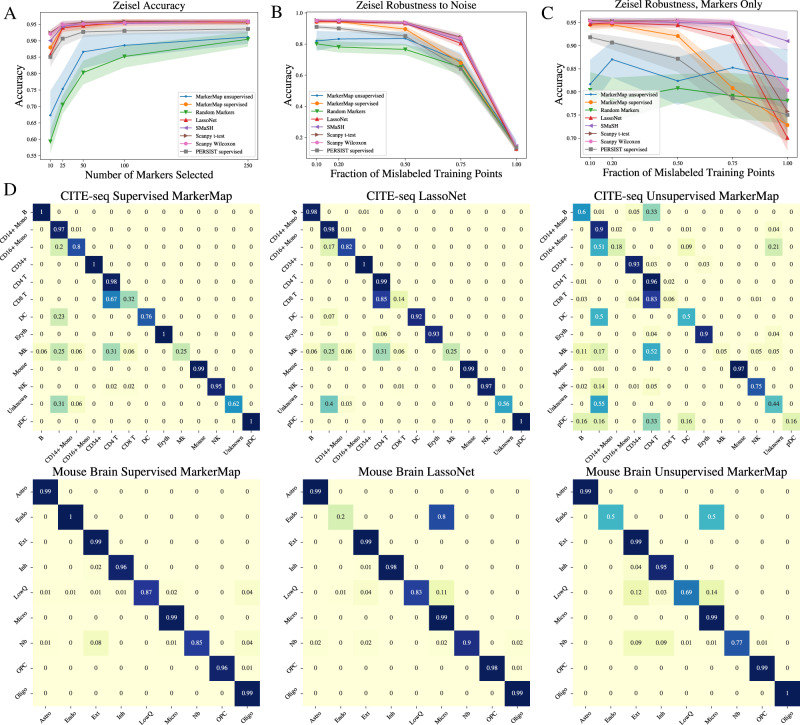
Predictive performance of MarkerMap. MarkerMap improves cell type prediction accuracy when annotations are provided (supervised setting) and allows for gene expression profile reconstructions when they are not (unsupervised and mixed settings). **A**–**C** Prediction accuracy of a variety of models on the Zeisel data set. **A** Accuracy increases as a function of the number of *k* markers selected, averaged over 10 runs. **B** Robustness to label noise, averaged over 10 runs, in the presence of classifiers trained on data with noisy labels, for *k* = 50 markers. **C** Robustness to misclassification when label errors are only present at the marker selection step, with classifiers trained on errorless data, averaged over 10 runs, for *k* = 50 markers. **D** Confusion matrices for Supervised MarkerMap, LassoNet, and Unsupervised MarkerMap on the CITE-seq and Mouse Brain data sets. Each method was restricted to the selection of 50 markers, and the classifier considered was a nearest neighbor classifier.

Next, we evaluated how the average accuracy varies with the target number of selected markers (Fig. 2). We find that MarkerMap performs particularly well in a low selected marker regime, with less than 10% marker selected. This may be particularly beneficial in applications like spatial transcriptomics where only a small number of genes can be tagged for observation. For calibration, we also included a set of random markers (that we report as baseline). The random set of markers performed rather well, outperforming two of the methods considered—Concrete VAE and Global-Gumbel VAE. We attribute the success of the random markers at classification to the high degree of correlation between features in biological studies. However, it is surprising that the sampling based baseline methods were outperformed by it.

MarkerMap is available in three variants—unsupervised, supervised, and joint (Table 1). Unsurprisingly, the supervised version performed best. The joint MarkerMap method was a close second, performing on par with the other top performers LassoNet and SMaSH. An attractive aspect distinguishing our method from SMaSH, in particular, is MarkerMap’s additional reconstruction loss. This allows learning markers that are both most predictive of cluster labels and best at reconstructing the full input data. This is particularly important in applications where feature collection is expensive or difficult. Finally, the unsupervised version of MarkerMap also had competitive performance. This version was trained without cluster information, hence suggesting that interpretable compression is possible for the biological study considered. When compared to approaches employing related sampling schemes—Concrete VAE and Global-Gumbel VAE, MarkerMap performs positively, suggesting that the differences in performance are largely due to parameter updating and aggregation across batches, rather than the sampling technique itself.

Interestingly, even though MarkerMap and LassoNet present comparable overall misclassification errors, the individual cluster misclassification values are quite different (Fig. 2). For example, in the CITE-seq data set, MarkerMap is slightly better at identifying the population of CD8 T and Eryth cells, while LassoNet is better at identifying the DC population and both methods have difficulties identifying Mk cells (Fig. 2). In contrast, Concrete VAE has a strong performance for a small set of cell types, but performs poorly in general compared to the other methods (Fig. 2). Likewise, in the Mouse Brain data set, MarkerMap is better at identifying endothelial cells (End) and low quality cells (LowQ), while LassoNet is better at identifying neuroblastoma cells (Nb). Given this, rather than advocating for a *best* method for this task, we instead advocate for transparent, easy to use, top performing methods, which can pick up different signals from the data.

### Learning representations which are robust to mislabeling

Further, we investigated the effects of mislabelled training data on MarkerMap and different benchmarks. Cell type labels often come from different processing pipelines and can be error prone. Hence, marker selection methods ought to show robustness when the training labels are not completely accurate.

To examine this effect we considered two experimental setups. First, we replaced the labels of a fraction of the training set by a random label uniformly sampled over all the possible training labels (Fig. 2). The misclassification rate was then measured only on the correctly labeled test data set. In this experiment, both the marker selection and the classifier were trained with incorrect labels so the performance decayed significantly when the fraction of misclassified points was large. Second, we also replaced the labels of a fraction of the training set by a random label uniformly sampled over all the possible training labels at the marker selection step, but the final classifier was trained on the correct labels on the (possibly incorrect) selected markers (Fig. 2). This experiment suggests all top performing methods (MarkerMap, LassoNet) are similarly stable with respect to noisy labels. The experiments also confirm that the performance of the unsupervised methods does not change, as they do not depend on input labels. Our numerical results are reported for the Zeisel, CITE-seq, Mouse Brain, Paul, and SSv4 data sets, and additional figures are presented in Supplementary Figures 3 through 14.

While the performance should be expected to deteriorate as the fraction of mislabelled training points increases, Fig. 2 shows that this happens *slowly* for small label noise. Recent theoretical results show that deep learning models can be robust to mislabelling^41–43^. This can be seen as a consequence of the consistency of certain estimators:^44^ shows this to be the case of a nearest neighbor classifier under general conditions. Such a margin is large enough to accommodate realistic expectations of mislabelling error in data sets; we do however note that there may be more complex, adversarial, or systematic sources of error for which robustness may not hold. Figure 2 echoes the good performance of a set of random markers, when the number of markers is sufficiently large^45^ and chosen to characterize a single cell type.

### Prospects for reconstruction in unsupervised settings

**Fig. 3:**
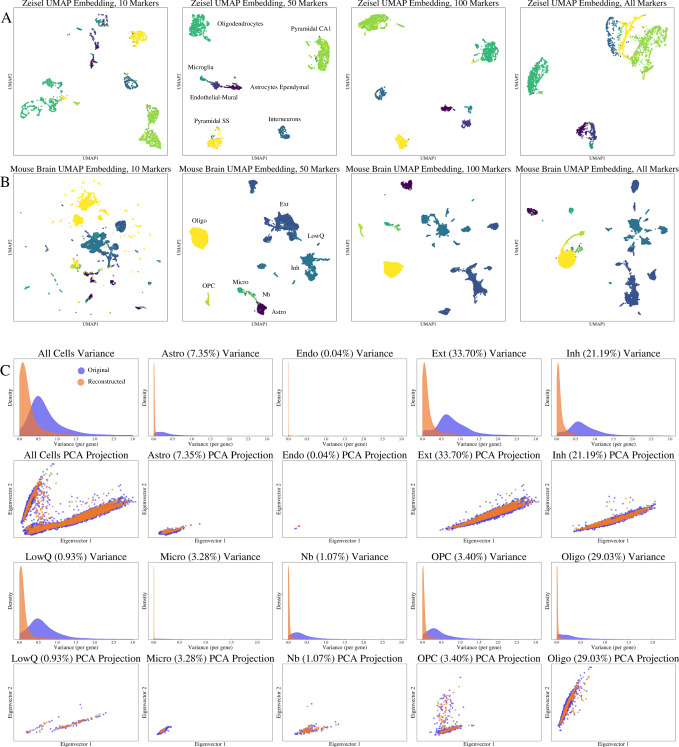
Downstream MarkerMap evaluation:visualization and reconstruction. **A** UMAP embeddings of the Zeisel data for different values of *k* markers. For UMAP, the parameters n_neighbors = 50, min_dist = 0.1 were used. **B** UMAP embeddings of the Mouse Brain data for different values of *k* markers. For all UMAPs, the parameters n_neighbors = 50, min_dist=0.1 were used. **C** In rows 1 and 3, histograms of gene expression variance values from the Mouse Brain data set for the original values and their corresponding reconstructions across cell types. In rows 2 and 4, PCA projections onto the first two eigenvectors of the original data along with their reconstructed counterparts. Additional variance and UMAP embedding figures are presented in the Supplementary Fig. 1 (variance plots), and Supplementary Figs. 2, 3, 4 (UMAP embeddings).

As a generative model, MarkerMap allows the reconstruction of the full transcriptomic input from the selected set of most informative markers. To understand the limits of this recovery, we first quantified the reconstruction quality by comparing distributional properties of the original and reconstructed data sets. Specifically, variances of genes from the reconstructed data were computed and compared to the variances of their counterparts in the original test data in a Mouse Brain data set, following unsupervised MarkerMap training with a 80–20% train-test split. The variances of the reconstructed data were lower than those of the original data (Fig. 3). This is a common phenomenon for generative models obtained with variational autoencoders, known as variance shrinkage^46,47^. To further visualize this, both test data and reconstructed data were projected onto the first two principle eigenvectors of the test data (Fig. 2).

We further assessed whether, despite variance differences, the highly variable genes in the original data are recapitulated in the reconstructed one. To this end, two metrics for relative ranking were employed: the Jaccard Index and Spearman Rank Correlation Coefficient, *ρ*. Additionally, average *ℓ*_2_ distance between the reconstructed expression profiles and the original expression profiles were computed per cell type (*Evaluation Metrics* and *Methods*).

Each of these metrics were computed for both the reconstructed data from MarkerMap and reconstructed data from a related generative model, scVI^48^. The scVI model learns the parameters of a zero-inflated negative binomial distribution for modeling genes counts from scRNA-seq data^48^. While both MarkerMap and scVI use a variational autoencoder framework for reconstruction, MarkerMap tries to reconstruct the full gene expression from the input of a small number of discrete markers, while scVI uses the full gene expression as input. In these experiments we used 50 markers for MarkerMap. Compared to scVI, MarkerMap generally scores worse on the variance metrics and better on the *ℓ*_2_ distance (Table 4). However, it should be noted that MarkerMap and scVI have slightly different goals that suggest that these results are appropriate. Unsupervised MarkerMap tries to find the best *k* markers that optimally reconstruct the full data, while the scVI model learns a low dimensional manifold from which data is generated. A direction of future exploration is leveraging the differential sampling scheme of MarkerMap and the generative power of scVI to improve MarkerMap’s reconstruction ability, while preserving its interpretability quality.

**Table 4:** Quality metrics for full transcriptome reconstruction

Cell types	MarkerMap Jaccard Index	Spearman *ρ*	*ℓ*_2_ Distance	scVI Jaccard Index	Spearman *ρ*	*ℓ*_2_ Distance
Astro	0.505	0.578	40.021	0.858	0.976	51.765
Endo	0.162	0.110	42.404	0.240	0.265	50.352
Ext	0.688	0.913	57.396	0.925	0.993	74.897
Inh	0.663	0.869	56.244	0.905	0.988	73.628
LowQ	0.551	0.722	52.773	0.690	0.859	66.035
Micro	0.340	0.351	35.379	0.762	0.945	44.239
Nb	0.351	0.438	42.272	0.634	0.848	53.200
OPC	0.465	0.591	45.875	0.794	0.952	58.249
Oligo	0.589	0.655	35.527	0.908	0.991	48.340
All	0.738	0.908	48.234	0.939	0.994	63.354

Reconstruction quality metrics comparing the original Mouse Brain data against data reconstructed from markers identified by MarkerMap, broken down by cell type and overall. The three left columns are for MarkerMap reconstruction and the right three columns are for scVI reconstruction. Each value is the averaged over 5 random splits of the training and testing data. For the Jaccard Index and Spearman *ρ*, higher scores are better, while for *ℓ*_2_ distance, lower scores are better. MarkerMap map uses 50 markers, while scVI uses the full 4581 genes.

## Discussion

In this work we propose MarkerMap, a data-driven, generative, neural network framework for feature selection. Given scRNA-seq data, we employ differentiable sampling methods to find a global set of genetic markers with competitive performance in downstream classification (of cell type) and reconstruction (of the entire transcriptome of an unseen test data). The supervised version selects the markers that maximize label prediction accuracy. The unsupervised version selects markers that maximize the reconstruction accuracy of a variational autoencoder (with no label information). A mixed MarkerMap is also available, combining both label prediction and transcriptome reconstruction. Our experiments suggest that, even though differentiable sampling techniques based on properties of the Gumbel distribution are often suggested for interpretable machine learning tasks, they can underperform. Hence, the mathematically appealing, continuous relaxation procedure alone is not enough to explain why MarkerMap is competitive with respect to alternatives. Additional exploration, both experimental and theoretical, is required to understand this empirical result. In this work, we provide a competitive solution to feature selection in a real biological context. Most importantly, we provide a tool where related solutions from different fields can be compared to aid future research in this area. A promising future application of this tool is the design of probes for spatial trascriptomics studies.

We provide an extensive numerical benchmark of both supervised and unsupervised tools in the context of genetic marker selection on real single cell gene expression data sets. We show that while all methods exhibit better performance as the number of selected markers increases, the methods have differences in stability when presented with noisy labels. The baselines considered originated from different research communities, which have not been previously compared to one another despite addressing similar tasks.

MarkerMap introduces new concepts from explainable machine learning in a transcriptomic centric setting. We show that MarkerMap is competitive across real data sets, thus offering the potential for optimal combinatorial experimental design with downstream analysis in mind. MarkerMap is available as a pip installable python package that is easy to use, robust and reproducible, making it appropriate for the experimental design of transcriptomic studies, along with the development of new metrics and methodology.

As deep generative models inspired by the growing explainability literature^27,28^ and foundation models literature become popular in genomics^37^, we sought to establish benchmarks for exploring both the potential and limitations of such tools, and thus included them in our analysis. Our message is simple: the flexibility of generative models can, in principle, improve both clustering and imputation, despite the need for more computational resources. This is increasingly the case for larger datasets, with a larger number of clusters and richer subclusters. Even if the improvements are small, they could be crucial in cases where rare cell types exist.

However, we saw a large variability in the performance of the different generative models considered, even as they share architectural similarities (e.g.,^37^ can perform worse than Scanpy subroutines on small datasets). Documenting such behaviors is crucial as the architectures of generative models become more involved. However, this skepticism should not temper the enthusiasm for generative model research: having access to good generative models means the ability to generate counterfactual data and to simulate perturbational scenarios in both spatial and non-spatial settings. While this lies outside the scope of our current paper, we hope to expand this exploration in follow-up work.

## Methods

### MarkerMap

MarkerMap is a generative method which belongs to the class of differentiable sampling techniques for subset selection^26–28^. Existing differentiable sampling techniques aim to find local features that suit each input individually. These methods have been used for and are relevant to language contexts where the input is usually a sequence of variable length representing text. For example, in an online market setting, we might want to learn what specific words or group of words of a review are most predictive of the score associated with the review. Instead, MarkerMap seeks to find a global set of features (markers when referring to genes), amenable to the structure of scRNA-seq data, which results in optimization differences.

In a nutshell, given high dimensional data points or gene expression profiles ${\{{x}_{i}\}}_{i = 1}^{n}\subset {{\mathbb{R}}}^{d}$, arranged in a matrix $X\in {{\mathbb{R}}}^{n\times d}$, the feature selection problem aims to find a subset of coordinates (i.e., markers, genes) *S* ⊂ {1, …, *d*}, ∣*S*∣ = *K*, relevant to a given downstream task (i.e., clustering, visualization, reconstruction). For example, in sparse linear regression, data *X* is used to predict responses $Y\in {{\mathbb{R}}}^{n}$ so that *Y* ≈ *Xβ* when only a small subset of the columns making up *X* is relevant for the prediction. Similarly, in non-linear settings, the search is over a joint pair (*β*, *f*), where *f* is a non-linear function so that *Y* ≈ *f*(*Xβ*).

Instead of optimizing for *β*, differentiable sampling methods assume informative samples are generated from a continuous distributions over a simplex with dimension equal to *K*, the number of features to be selected^26–29^. This is accomplished through a selector layer. In detail, the selector layer contains *k* = 1, …*K* nodes. The nodes are associated with a *d*-dimensional real-valued vector *γ*^(*k*)^ which governs the probability that a feature will be selected, whose entries *j* are equal to:

1 $$\begin{array}{r}{\gamma }_{j}^{(k)}=\frac{\exp \left(\left(\log \left({\pi }_{j}^{(k)}\right)\,+\,{g}_{j}^{(k)}\right)/\tau \right)}{\mathop{\sum }\nolimits_{s = 1}^{d}\exp \left(\left(\log \left({\pi }_{s}^{(k)}\right)\,+\,{g}_{s}^{(k)}\right)/\tau \right)},\end{array}$$

where ${g}_{j}^{(k)}$ are independent samples from a Gumbel distribution with location 0 and scale 1, *τ* is positive and real, and *π*^(*k*)^ represent the class probabilities over a categorical distribution. The *γ*^(*k*)^ is a vector following a Gumbel-Softmax distribution, independently introduced by^29^ and^26^. This distribution takes the form

2 $${p}_{\pi ,\tau }({\gamma }^{(1)},...{\gamma }^{(K)})=(K-1)!{\tau }^{K-1}{\left(\mathop{\sum }\limits_{i = 1}^{K}\frac{{\pi }^{(i)}}{{\left({\gamma }^{(i)}\right)}^{\tau }}\right)}^{-K}\mathop{\prod }\limits_{i=1}^{K}\left(\frac{{\pi }^{(i)}}{{\left({\gamma }^{(i)}\right)}^{\tau +1}}\right),$$

and can be visualized over the (*K* − 1)-dimensional simplex.

The number *τ* is referred to as temperature and the values $\log {\pi }^{(k)}$ are called logits. The logits control how likely a feature or gene *j* is likely to be selected as a representative feature *k* our of the total *K* features we can select. For an input ${x}_{i}={({x}_{ij})}_{j = 1}^{d}$, each node *k* of the selector layer outputs ${x}_{i}\,*\,{y}^{(k)}$, which essentially masks the genes that are deemed uninformative. As the temperature *τ* approaches 0, $Pr({\gamma }_{j}^{(k)}=1)\to {\pi }_{j}^{(k)}/{\sum }_{s}{\pi }_{s}^{(k)}$, and only one feature of *x*_*i*_ is selected and matched with a unique selector node *k*^28^.

### Illustrative Toy Example

Consider a data set of $X={\{{x}_{i}\}}_{i = 1}^{n}$ gene expression profiles, corresponding to *n* cells with 10,000 variable genes. In this toy scenario, we only record if these gene are overexpressed (+1) or under expressed (-1) with respect to some control population. A natural task would be to attempt to compress our data by expressing it in a lower dimension, which is often achieved with a VAE (*Architecture*). The cells come in two states *A* and *B*, depending on how they respond to a particular perturbation, a response which we observe. Assume that whether the cells are in state *A* or state *B* only depends on 3 genes in the following way. A cell is in state *A* if the genes are either all overexpressed (1,1,1) or all underexpressed (-1,-1,-1), otherwise they are all in cell state *B*. None of the genes are individually informative of the clustering, the mean per cluster would be 0 for all genes. Initially, we may not know which of the 10,000 genes are indicative of the cell states, but we’d like to obtain a reduced number of genes capable of accurately predicting cell state. For example, without prior information, one might assume all genes are equally good at this task, so our initial ‘weighting’ of the genes would be ($\frac{1}{10,000},\frac{1}{10,000},\ldots \frac{1}{10,000}$) (*Parameter Initialization*) which would translate to a continuous probability distribution through equation (1) i.e. they specify the *π*_*j*_. The uniformity corresponds to randomly picking any of the genes as informative features. Then, through the variational optimization algorithm (*Optimization*), these weights are iterated on until, upon convergence, the genes that are jointly more indicative of cell state will have a higher probability weight, while also being sufficient for the reconstruction of the entire gene expression. This will correspond to a vector ($\frac{1}{3},\frac{1}{3},\frac{1}{3},0,0\ldots 0$) assuming the first three genes are the most informative.

### Optimization

Letting *p*(*x*) be the probability distribution over the *d*-dimensional data *X* and given a set of labels *Y*, MarkerMap learns: a) a subset of markers *S* of size *K*, b) a reconstruction function ${f}_{\theta }:{{\mathbb{R}}}^{K}\to {{\mathbb{R}}}^{d}$, and c) a classifier ${f}_{W}:{{\mathbb{R}}}^{K}\to {{{\mathcal{Y}}}}$.

To learn these elements, the following empirical objective is optimized:

3 $$\begin{array}{r}\mathop{{\mathrm{arg}}\,{\mathrm{min}}}\limits_{S,\theta, W}{{\mathbb{E}}}_{p(x)}[\parallel {f}_{\theta }({x}_{S})-x{\parallel }_{2}+\ell ({f}_{W}({x}_{S}),y(x))],\end{array}$$

where the first term optimizes signal reconstruction from a subset of markers *x*_*S*_ and the second objective minimizes the expected classification risk, both over the unknown distribution *p*(*x*) with respect to a loss function *ℓ*. In practice, we consider the alternative empirical objective

4 $$\begin{array}{r}\mathop{{\mathrm{arg}}\,{\mathrm{min}}}\limits_{S,\theta, W}\,\alpha \parallel {f}_{\theta }({X}_{S})-X{\parallel }_{2}+(1-\alpha )\parallel ({f}_{W}\,({X}_{S}),Y){\parallel }_{2},\end{array}$$

where *α* ∈ [0, 1] serves to balance between a reconstruction loss and classification loss. MarkerMap considers three separate objectives: a supervised objective with *α* = 0, an unsupervised objective with *α* = 1, and a joint objective where *α* = 0.5. More generally, *α* can be treated as a tunable (but fixed) hyperparameter that weighs the reconstruction and classification terms in the optimization objective. Because full reconstruction is nominally a harder task it can be considered a bottleneck, since one can achieve low classification error without information about the entire gene expression. Thus, when *α* is small enough, the convergence of MarkerMap is dependent on the quality of the reconstruction. Depending on the user-specified goal, the three proposed values of *α* provide either a classifier (*α* = 0) which may be capable of selecting a smaller number of genes with good performance, a generative model (*α* = 1) which is capable of signal reconstruction possibly at the cost of additional markers needed, or both (*α* = 0.5). One may choose a different value of *α* that is possibly data- or problem-specific.

Optimizing this objective is difficult due to the combinatorial search over the subset *S*. We address this challenge heuristically by expanding on continuous sampling techniques^27^ in a batch learning setting^49^. In a nutshell, *b* = 1, 2, …*B* batches are sampled without replacement from the data set (*X*, *Y*). The selected features are then computed and aggregated across batches as follows:

1. Instance-wise logits ${{{{{{\rm{log}}\; \pi }}}}}_{i}^{b}={f}_{\pi }({x}_{i})$ are generated for each *x*_*i*_ in the batch *b*, where *f*_*π*_ is a neural network. Averaging them leads to an intermediate average batch logit log π^*b*^.
2. The average batch logits are computed by aggregating information from the current and previous batches, $\log {\pi }^{b}\leftarrow \beta {{{{{{\rm{log}}\; \pi }}}}}^{b-1}+(1-\beta ){{{{{{\rm{log}}\; \pi }}}}}^{b},\beta \in (0,1)$ much like the update for mean moment in BatchNorm^49^.
3. The *K* continuous *d*-dimensional hot encoded vectors ${\gamma }^{(k),b}={({\gamma }_{j}^{(k)})}_{j = 1,d}^{b}$ are generated from $\log {\pi }^{b}$ via continuous relaxation, see (1).
4. Each *γ*^(*k*),*b*^ selects one of the *K* features by element-wise multiplication ${X}_{S}^{b}={X}^{b}\boxtimes {\gamma }^{b}$.
5. The resulting ${X}_{S}^{b}$ then becomes the input in a Variational-Autoencoder-like architecture, which includes a classifier loss as well as a reconstruction (Fig. 1 and Eq. (4)).
6. All network weights are updated through stochastic gradient descent steps, following the optimization of the appropriate loss in Eq. (4) until convergence. The steps are repeated for *B* timesteps, corresponding to the number of batches.

### Architecture

The three main components of MarkerMap’s architecture are the neural network *f*_*π*_ for instance-wise logit generation, the task specific feed-forward network *f*_*W*_ for classification, and the variational autoencoder *f*_*θ*_ for encoding and reconstruction. The neural network *f*_*π*_ is an encoder with two hidden layers and a sampling layer performing relaxed subset sampling^27^. For supervised tasks, *f*_*W*_ is represented by a decoder with one hidden layer. The encoder component of the variational autoencoder *f*_*θ*_ has two hidden layers, while the Gaussian decoder has one hidden layer. All the hidden layers have the same size and are data set dependent, except for the Gaussian latent layer which has dimension 16 across experiments. The activation functions were chosen as follows: Leaky Rectified Linear Unit functions for hidden layers, identity transformation for the last layer of *f*_*θ*_ and softmax for the last layer of *f*_*W*_. All activations were preceded by batch normalization in all hidden layers to mediate vanishing gradients.

### Temperature annealing

The temperature *τ* in (1) is a key parameter in the sampling procedure. It controls how fast the continuous encoding vectors *γ*^(*k*)^ approach a true one-hot encoding. Low values of *τ* emulate true feature selection, while higher values of *τ* are more likely to extract linear combinations of features. However, 0 < *τ* < 1 leads to inconsistent feature selection^27^. To mediate this issue, we used a temperature annealing scheme. First, we initialize *τ*_initial_ > 1. This leads to gradients with less batch to batch variability and more diversity in feature selection, as *γ*^*b*^ will be more diffuse. Second, we decay the temperature during training by a constant factor^28^. We found that setting *τ*_initial_≥2 with a decay factor leading to a *τ*_final_ ∈ (0.001, 0.1) resulted in good performance across all experiments.

### Parameter initialization

MarkerMap allows us to initialize the logits log π^*b*=0^ with an informed guess of which markers are relevant. In the absence of prior information we initialize the logits as log π^*b*=0^ = **1***c*, where *c* is any constant. The weights of each linear layer are initialized using Kaiming initialization^50^. The weights of the BatchNormalization layers are initialized as a vector of **1** for scaling and a vector of **0** for the biases.

For backpropagation we use the Adam optimizer with a learning rate obtained via a learning rate finder^51^. A range of learning rates between 1e-8 and 0.001 are explored in linear intervals, with a minimum of 25 epochs and max of 100 epochs. Training can end early when the average loss on the validation set does not decrease after 3 epochs.

In all our experiments we randomly split the data in training (70%), validation (10%), and test sets (20%). The batch size is 64 for all data sets. The quality of the markers selection did not depend on batch size (with tested values of 32, 64, and 128 on the Zeisel and Paul data sets). For the hidden layer size, we chose values approximately equal to $\frac{1}{10}{{{\rm{th}}}}$ the number of genes in each data set. This heuristic showed positive empirical results, while also keeping the network to a reasonable size. This resulted in a hidden layer size of 256 for Zeisel and Paul, 64 for CITEseq, and 500 for Mouse Brain and SSv4.

### Scalability

Training MarkerMap on the 4581 genes and 39,583 cells of the Mouse Brain data set (the largest data set considered) on public cloud GPUs resulted in a training time of 5 minutes for supervised classification tasks, and 15 minutes for unsupervised tasks. LassoNet performed similarly when the architecture (number of hidden layers and units) and batch sizes were chosen to be similar to those of MarkerMap. RankCorr and SMaSH achieved smaller training times, less than a minute, but require supervised signals. The differential expression tests in Scanpy and COSG are quick but also require supervised signals. PERSIST benefits somewhat by taking a two step approach to learning markers, but the initial step makes the method take longer.

### Benchmarks

We contrast MarkerMap against several subset selection methods. The methods have been introduced in different communities and many have not been previously compared to one another.

- LassoNet: A residual feed-forward network that makes use of an *ℓ*_1_ penalty on network weights in order to induce sparsity in selected features^25^.
- Concrete VAE: a traditional VAE architecture that assumes a discrete distribution on latent parameters and performs inference using the formulation of the concrete distribution (also known as Gumbel-Softmax distribution)^26^.
- Global-Gumbel VAE: adapted from^27^. A VAE architecture related to the Concrete VAE.
- Smash Random Forest: A classical Random Forest classification algorithm implemented in the SMaSHpy library (see https://pypi.org/project/smashpy)^16^.
- RankCorr: A non-parametric marker selection method using (statistical) rank correlation, implemented in the RankCorr library (see https://github.com/ahsv/RankCorr)^15^.
- PERSIST: An autoencoder model similar to MarkerMap that finds markers in an unsupervised fashion, or uses cell labels for supervised learning. PERSIST uses specific loss functions geared towards scRNA-seq data and a two step process to find the most relevant markers. (see https://github.com/iancovert/persist/)^37^
- COSG: A differential expression test based on cosine similarity of expression of different genes. (see https://github.com/genecell/COSG)^38^.
- Scanpy: The ‘rank_genes_groups` function of this package performs differential expression tests based on the cell groups with a number of different statistical methods. We tested the methods *t*-test, *t*-test with overestimated variance, Wilcoxon-ranked sum test, and Wilcoxon-ranked sum test with tie correction (see https://scanpy.readthedocs.io/en/stable/index.html)^39^
- Scanpy Highly Variable Genes: The ‘highly_variable_genes’ function of this package^39^ performs an unsupervised method from Seurat^52^ to select genes that are highly variable.

The differential expression tests are one-vs-all methods. For these, we took one marker from each cell type, removing duplicates, until that would put us over our budget *k*. Then we took the marker with the highest score (COSG) or lowest *p*-value (Scanpy) until we had *k* markers.

### Data sets

We used publicly available real world data sets from established single cell analysis pipelines, where the problem of marker selection is of interest in the context of explaining cluster assignment. In each data set, the labels correspond to cell types.

#### Zeisel data set

The Zeisel data set contains data from 3005 cells and 4000 genes^32^. The cells were collected from the mouse somatosensory cortex (S1) and hippocampal CA1 region. The labels correspond to 7 major cell types and where obtained though biclustering of the full gene expression data set. For the Zeisel (subtypes) data set, we used the more specific 47 cell types. We removed cells whose specific cell types were unknown, leaving 2816 cells.

#### CITE-seq data set

Cellular Indexing of Transcriptomes and Epitopes by Sequencing (CITE-seq) is a single cell method that allows joint readouts from gene expression and proteins. The CITE-seq data set contains data from 8617 cells and 500 genes^33^. These cells correspond to major cord blood cells across 13 cell types, obtained from the clustering of combined gene expression and protein read-out data, and not from the clustering of the original single cell data set alone.

#### Paul data set

The Paul data set^35^ consists of 2730 mouse bone marrow cells, collected with the MARS-seq protocol. Post processing, each cell contains 3451 genes. The Paul data set contains progenitor cells that are differentiating, hence the data appear to follow a continuous trajectory. The associated outputs represent 10 discrete cell types sampled along these trajectories. Hence, the cell types are are not well separated^35^. After removing general genes and housekeeping genes, we are left with 3074 genes. For this data set we do not further remove genes based on cell type because the data set is already small.

#### Mouse brain data set

This data set is a spatial transcriptomic data set, containing data from 40,572 cells and 31,053 genes from diverse neuronal and glial cell types across stereotyped anatomical regions in the mouse brain^34^. The output labels correspond to the major cell types identified by the authors. Prior to the pre-processing described below, we perform additional gene and cell filtering because training with the full data set was not feasible for the unsupervised model on public cloud infrastructure. We start by removing cells with unknown cell types. Then we keep only those genes that satisfy the following two conditions: (1) they are present in at least 0.05% of cells and (2) they are present in 3% of cells or the average gene expression level in cells where the gene is present is greater than 1.12. These particular values are somewhat arbitrary and could be changed based on the researcher’s desires. After this filtering we are left with 39,583 cells and 12,869 genes; further pre-processing described below will reduce the number of genes to 4581. In the Mouse Brain data set we use the 9 major cell types after removing those that are unknown. In the Mouse Brain (subtypes) data set we use all 59 specific cell types, which includes two different unknown categories. When leaving these two unknown categories in, we are working with 40,532 cells and 7115 genes after the pre-processing described below.

#### SSv4 V1 data set

The SSv4 data set^36^ consists of cells collected from the mouse primary visual cortex (V1). This publicly available data set includes initial pre-processing done by PERSIST^37^ which reduces the data set to 13,349 cells and 10,000 genes with 98 cell types. We removed 6 cell types that each had fewer than 4 cells because many of our supervised methods require multiple representatives per class. After further pre-processing described below, the resulting data set size is of 13,342 cells and 4293 genes.

### Data processing

The data were processed and filtered following^16,33^. In particular, we first remove genes associated with general cell function as well as housekeeping genes. Next, we remove genes which are in present in less than 30% of cells for every cell type. We also remove genes which are present in over 75% of cells for at least 50% of the cell types. Lastly, we normalize the gene counts per cell so that each cell has the same total gene expression, we perform a ${\log }_{2}(1+x)$ transform of the cell counts, and we center and scale the data so that each gene has mean 0 and variance 1. When evaluating the generative data, we forgo normalizing gene counts across cells and setting the mean to 0 and the variance to 1 of each gene. Instead, we only perform the log_2_(1 + *X*) transform and then set the mean and variance of the entire data matrix *X* to 0 and 1 respectively.

### Evaluation metrics

Given *K*, most of the methods selected the top *K* features informative of ground-truth labels. The exceptions, RankCorr and LassoNet, do not allow the selection of an exact number of features, as they rely on specifying a regularizer parameter that controls feature sparsity. In those cases, we selected *K* features by grid searching the regularizer that would get the desired number of features.

For each baseline and data set, the selected features were then used as only input to a either a nearest neighbors classifier or a random forest classifier. For each data set, method and classifier type, we reported two quantities, the misclassification rate and a weighted F1 score, along with their corresponding confusion matrices. These quantities are defined as follows, for a number of ground truth clusters *c* = 1, 2, …*C*.

- *Average misclassification rate*. The misclassification rate of a given cluster is defined as
  5 $$\begin{array}{r}{M}_{c}=1-\frac{T{P}_{c}}{T{P}_{c}\,+\,F{P}_{c}},\end{array}$$
  where TP and FP correspond to the number of true positives and false positive predictions, respectively. We report the average misclassification $\frac{1}{C}{\sum }_{c}\,{M}_{c}$.
- *Average F1 score*. Per cluster, the F1 score is defined as
  6 $$\begin{array}{r}{F}_{c}=\frac{2{P}_{c}{R}_{c}}{{P}_{c}\,+\,{R}_{c}},\end{array}$$
  where *P*_*c*_ and *R*_*c*_ are the precision and recall of the classifier for a cluster *c*. We report the average F1 score $\frac{1}{C}{\sum }_{c}\,{F}_{c}$.

When evaluating the reconstructed data, we use the Jaccard Index, the Spearman Correlation Coefficient *ρ*, the *ℓ*_2_ distance, and the *ℓ*_1_ distance. Let $X\in {{\mathbb{R}}}^{n\times d}$ be our data as before, and let $\tilde{X}\in {{\mathbb{R}}}^{n\times d}$ be the reconstructed data.

- *Jaccard Index*. First we calculate the variances of each gene in the original data. Since each gene is a column of *X*, the variance of those columns is a *d*-length vector which we will denote ${\sigma }_{X}^{2}$. Next we find the rank vector of the variances, $R({\sigma }_{X}^{2})$, where the largest variance is assigned 1, the second largest is assigned 2, and so on until the smallest variance is assigned *d*. We use the ranks to find the indices of the largest 20% of the variances:
  7 $${I}_{X}=\left\{i:R\left({\sigma }_{X}^{2}\right)[i]\le \frac{d}{5}\right\}$$
  We follow the same process for the reconstructed data to get the set of indices ${I}_{\tilde{X}}$. Finally, we calculate the Jaccard Index on these two sets of indices to determine their similarity^53^:
  8 $$J=\frac{\left\vert {I}_{X}\cap {I}_{\tilde{X}}\right\vert }{\left\vert {I}_{X}\cup {I}_{\tilde{X}}\right\vert }$$
  The Jaccard Index ranges from 0 to 1, and higher values indicate that more of the highly variable genes from the original data are also highly variable in the reconstructed data.
- *Spearman correlation coefficient*. The Spearman correlation coefficient is exactly the Pearson correlation coefficient calculated on the ranks of a vector’s values, rather than the raw values. Thus, we first calculate the rank vectors of the gene variances as we did for the Jaccard Index, $R({\sigma }_{X}^{2})$ and $R({\sigma }_{\tilde{X}}^{2})$. Finally we calculate the correlation coefficient:
  9 $$\rho =\frac{\,{{\mbox{cov}}}\,\left(R\left({\sigma }_{X}^{2}\right),R\left({\sigma }_{\tilde{X}}^{2}\right)\right)}{{\sigma }_{R\left({\sigma }_{X}^{2}\right)}{\sigma }_{R\left({\sigma }_{\tilde{X}}^{2}\right)}}$$
  where ${\sigma }_{R({\sigma }_{X}^{2})}$ and ${\sigma }_{R({\sigma }_{\tilde{X}}^{2})}$ are the standard deviations of the ranks of the original data and the reconstructed data respectively. This *ρ* is the Spearman correlation coefficient—values closer to one indicate higher similarity of the ranks of the gene variances.
- *ℓ*_2_ *Distance*. To calculate the *ℓ*_2_ distance, we take the average over all cells of the *ℓ*_2_ distance between the original cell and the reconstructed cell:
  10 $$\frac{1}{n}\mathop{\sum }\limits_{i=1}^{n}\parallel {x}_{i}-{\tilde{x}}_{i}{\parallel }_{2}$$
  where *x*_*i*_ is the *i*^*th*^ row of *X*. Lower values indicate that the original data and reconstructed data are more similar.
- *ℓ*_1_ *Distance*. To calculate the *ℓ*_1_ distance, we take the average over all cells of the *ℓ*_1_ distance between the original cell and the reconstructed cell:
  11 $$\frac{1}{n}\mathop{\sum }\limits_{i=1}^{n}\parallel {x}_{i}-{\tilde{x}}_{i}\parallel$$
  where *x*_*i*_ is the *i*^*th*^ row of *X*. Lower values indicate that the original data and reconstructed data are more similar.

### Reporting summary

Further information on research design is available in the Nature Research Reporting Summary linked to this article.

### Supplementary information

Supplementary Figures

Reporting summary

## Data Availability

We used publicly available data as detailed in the Data sets section.

## Code availability

The code is available as a Python package at https://github.com/Computational-Morphogenomics-Group/MarkerMapand on pip as “markermap”. See Fig. 1 for an overview of the package functionality. Code to easily load and pre-process the four data sets used in this paper are provided. Additional pre-processing can be done with the Scanpy package, and MarkerMap also provides functions to manage to split the data into training and test sets. The package implements MarkerMap as well as Concrete VAE and Global Gate VAE. Additionally, it provides wrappers for all the other mentioned methods to allow for easy benchmarking. All models select *k* markers, which are then used for further tasks including visualizations.
