# Supplementary material for: MarkerMap: nonlinear marker selection for single-cell studies
Source: NPJ Syst Biol Appl. 2024 Feb 14;10:17. doi: 10.1038/s41540-024-00339-3 (PMC10864304; doi:10.1038/s41540-024-00339-3)
Supplement: Supplementary file 1 — Supplementary Figures [file 41540_2024_339_MOESM1_ESM.pdf]

Supplementary Information for MarkerMap

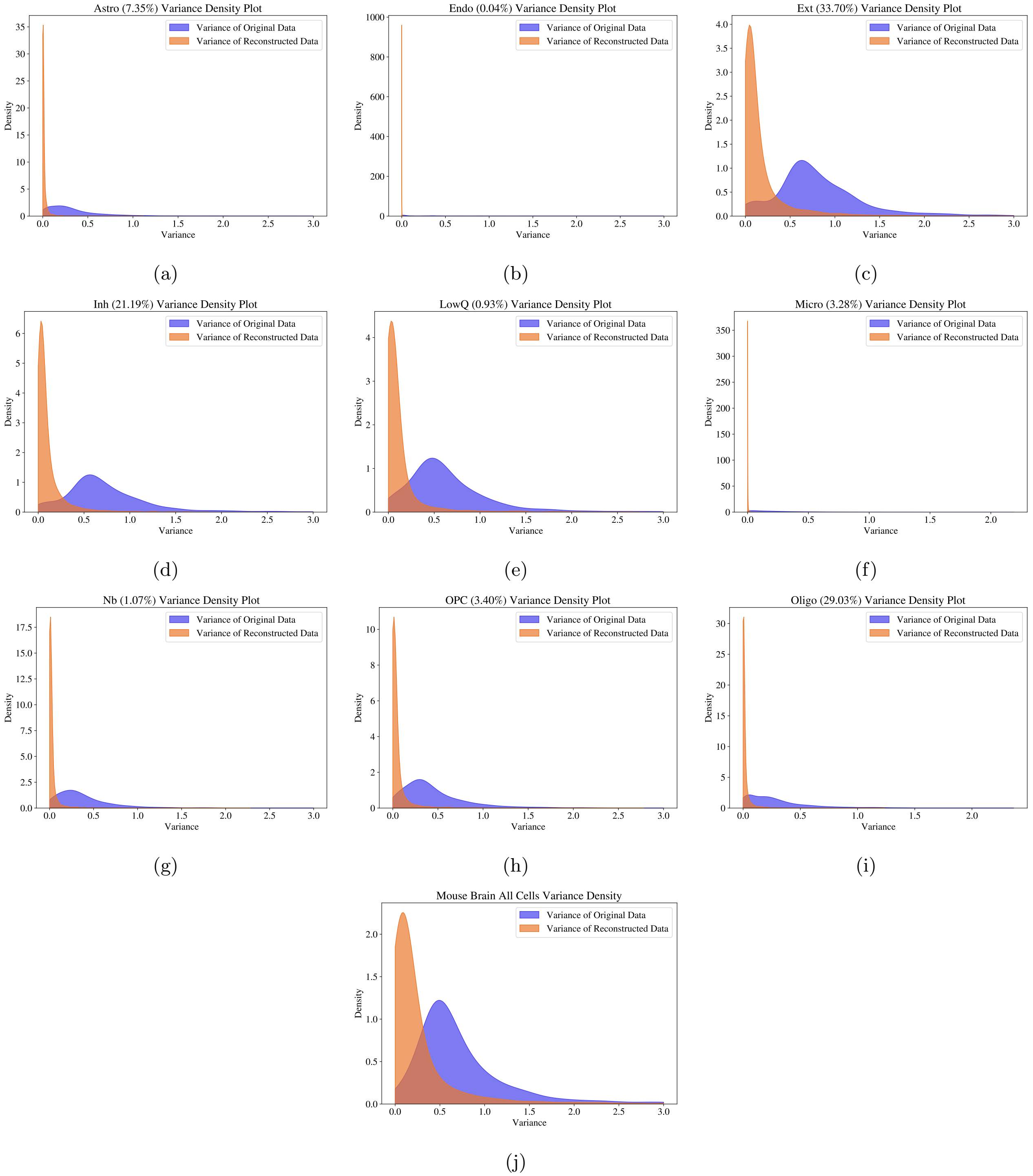

Supplementary Figure 1: Density plot of variances of gene expression levels for the original data and the reconstructed data of all classes of cells from a test set of the Mouse Brain data. The percents represent how many cells of that type are in the test set. Panel (a): Astro (7.35%) class. Panel (b): Endo (0.04%) class. Panel (c): Ext (33.70%) class. Panel (d): Inh (21.19%) class. Panel (e): Low Quality cells (0.93%) class. Panel (f): Micro (3.28%) class. Panel (g): Nb (1.07%) class. Panel (h): OPC (3.40%) class. Panel (i): Oligo (29.03%) class. Panel (j): All cells.

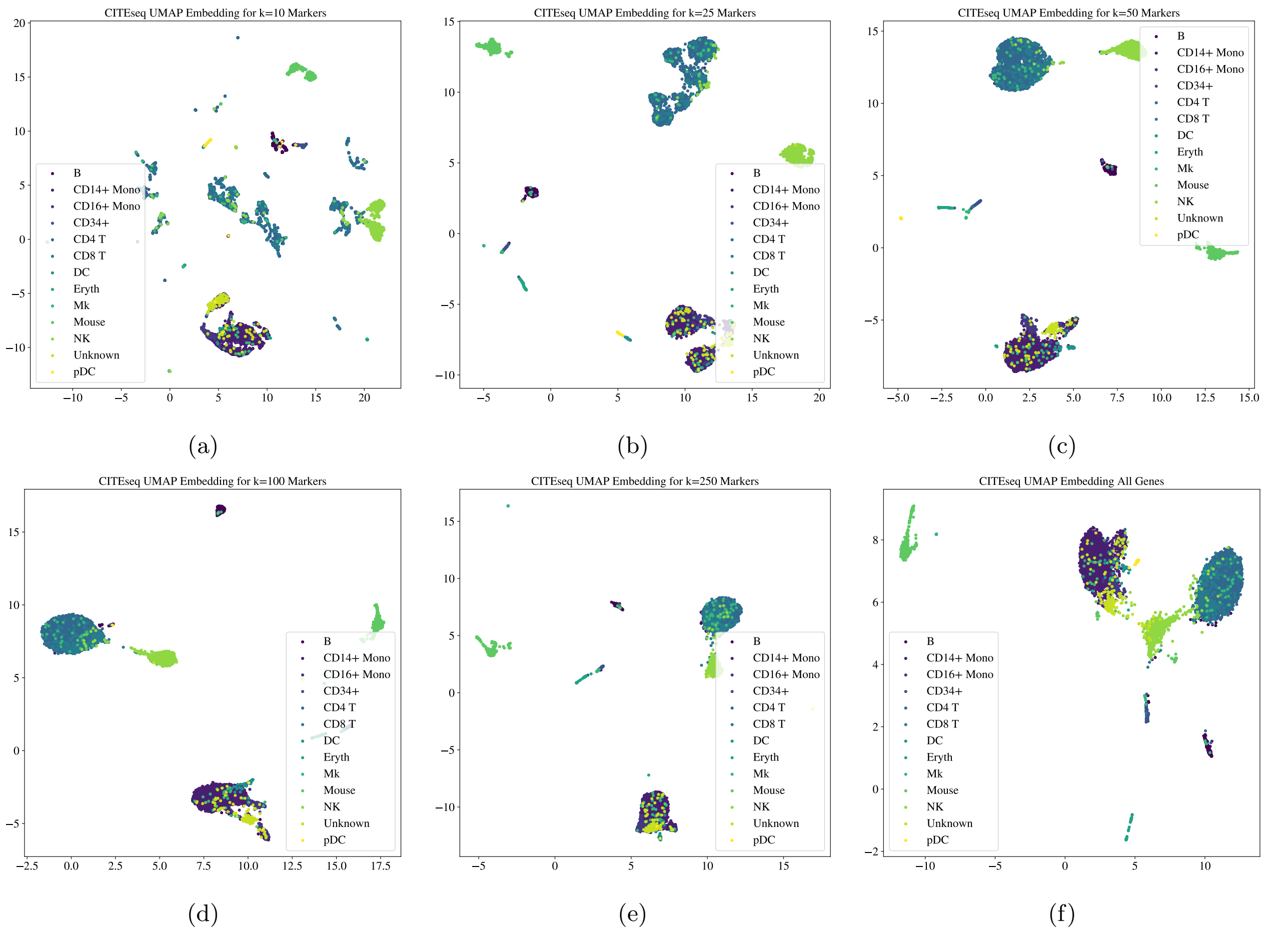

Supplementary Figure 2: Plots of UMAP embeddings for various levels of k for the CITEseq data set. Panels (a)-(e) show umap embeddings for $k = 10, 25, 50, 100, 250$ markers respectively, and panel (f) shows the umap embedding using all genes.

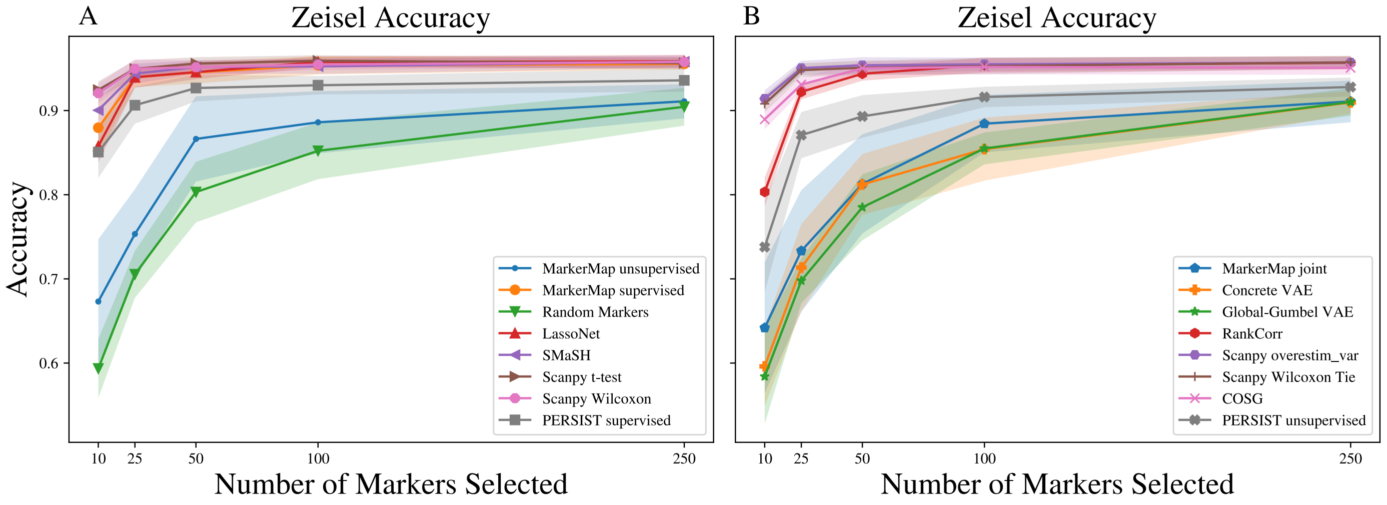

Supplementary Figure 3: Accuracy benchmark over k using a Random Forest classifier for the Zeisel data set, averaged over 10 runs. Panel **A**: Models MarkerMap unsupervised, MarkerMap supervised, Random Markers, LassoNet, SMaSH, Scanpy t-test, Scanpy Wilcoxon, and PERSIST supervised. Panel **B**: Models MarkerMap joint, Concrete VAE, Global-Gumbel VAE, RankCorr, Scanpy overestimate variance, Scanpy Wilcoxon with tie correction, COSG, and PERSIST unsupervised. Note that both panels use the same y-axis for direct comparison.

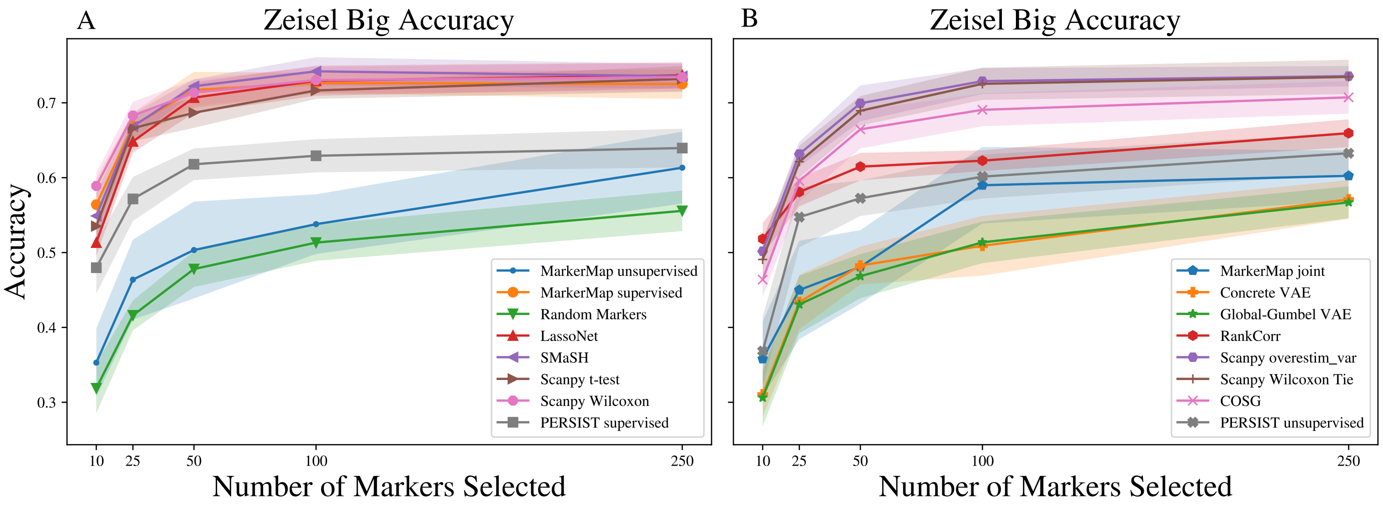

Supplementary Figure 4: Accuracy benchmark over k using a Random Forest classifier for the Zeisel Big data set, averaged over 10 runs. Panel **A**: Models MarkerMap unsupervised, MarkerMap supervised, Random Markers, LassoNet, SMaSH, Scanpy t-test, Scanpy Wilcoxon, and PERSIST supervised. Panel **B**: Models MarkerMap joint, Concrete VAE, Global-Gumbel VAE, RankCorr, Scanpy overestimate variance, Scanpy Wilcoxon with tie correction, COSG, and PERSIST unsupervised. Note that both panels use the same y-axis for direct comparison.

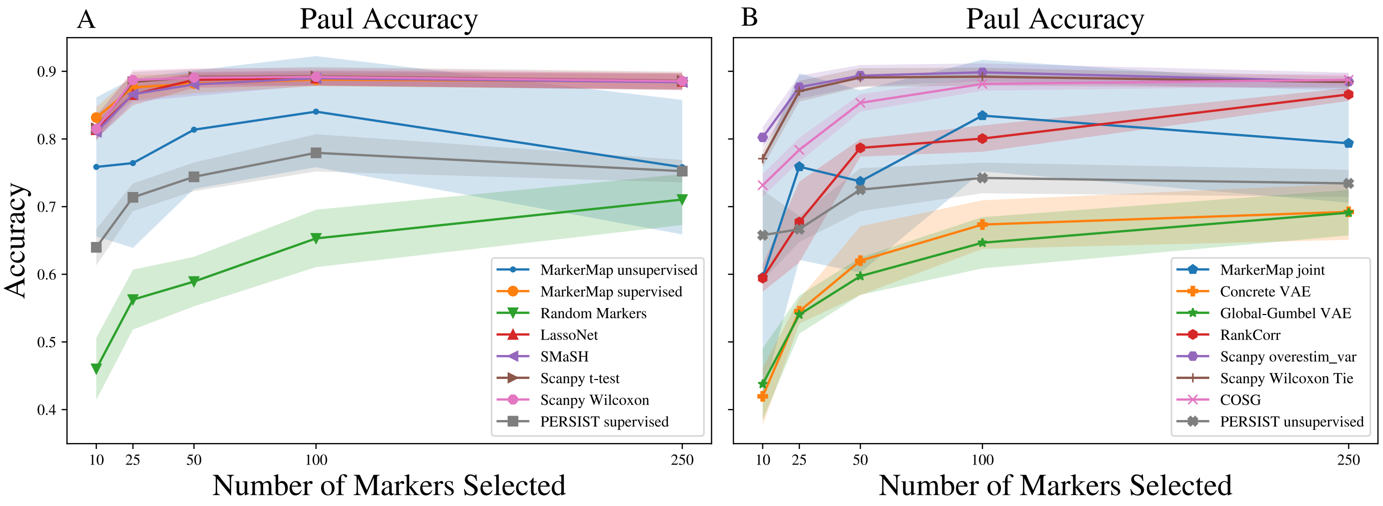

Supplementary Figure 5: Accuracy benchmark over k using a Random Forest classifier for the Paul data set, averaged over 10 runs. Panel **A**: Models MarkerMap unsupervised, MarkerMap supervised, Random Markers, LassoNet, SMaSH, Scanpy t-test, Scanpy Wilcoxon, and PERSIST supervised. Panel **B**: Models MarkerMap joint, Concrete VAE, Global-Gumbel VAE, RankCorr, Scanpy overestimate variance, Scanpy Wilcoxon with tie correction, COSG, and PERSIST unsupervised. Note that both panels use the same y-axis for direct comparison.

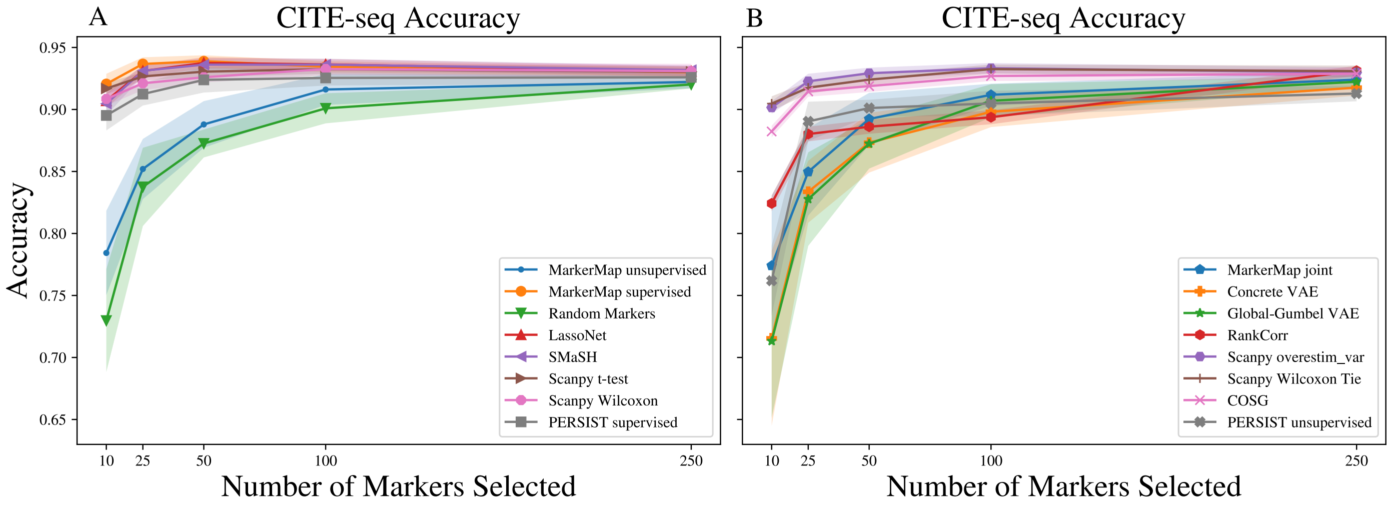

Supplementary Figure 6: Accuracy benchmark over k using a Random Forest classifier for the CITE-seq data set, averaged over 10 runs. Panel **A**: Models MarkerMap unsupervised, MarkerMap supervised, Random Markers, LassoNet, SMaSH, Scanpy t-test, Scanpy Wilcoxon, and PERSIST supervised. Panel **B**: Models MarkerMap joint, Concrete VAE, Global-Gumbel VAE, RankCorr, Scanpy overestimate variance, Scanpy Wilcoxon with tie correction, COSG, and PERSIST unsupervised. Note that both panels use the same y-axis for direct comparison.

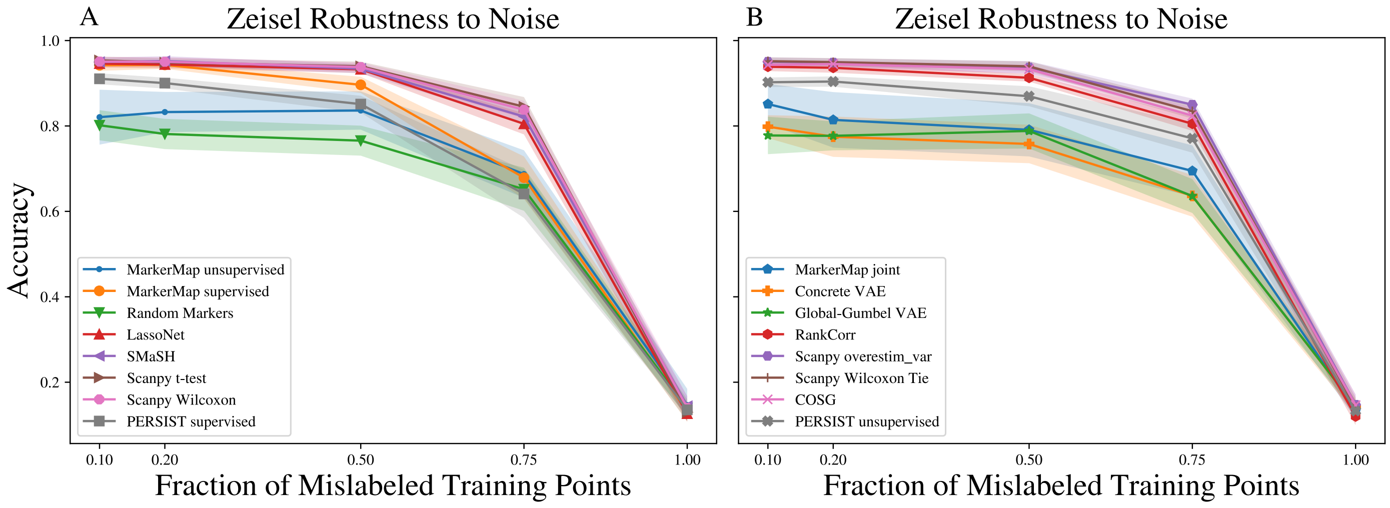

Supplementary Figure 7: Accuracy benchmark over label error using a Random Forest classifier for the Zeisel data set, averaged over 10 runs. Panel **A**: Models MarkerMap unsupervised, MarkerMap supervised, Random Markers, LassoNet, SMaSH, Scanpy t-test, Scanpy Wilcoxon, and PERSIST supervised. Panel **B**: Models MarkerMap joint, Concrete VAE, Global-Gumbel VAE, RankCorr, Scanpy overestimate variance, Scanpy Wilcoxon with tie correction, COSG, and PERSIST unsupervised. Note that both panels use the same y-axis for direct comparison.

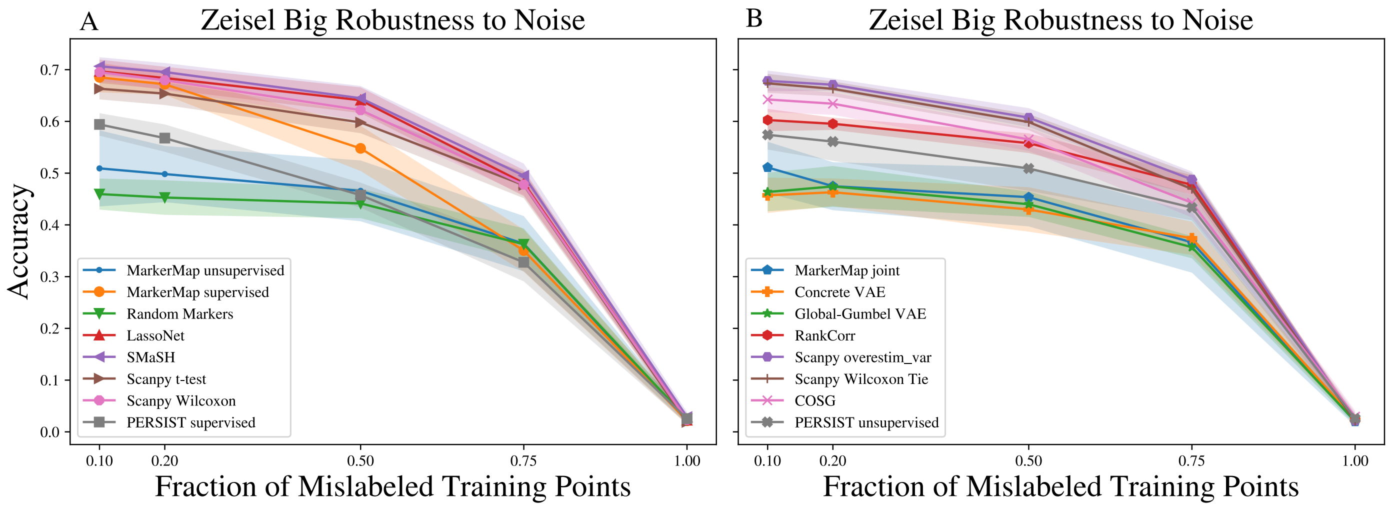

Supplementary Figure 8: Accuracy benchmark over label error using a Random Forest classifier for the Zeisel Big data set, averaged over 10 runs. Panel **A**: Models MarkerMap unsupervised, MarkerMap supervised, Random Markers, LassoNet, SMaSH, Scanpy t-test, Scanpy Wilcoxon, and PERSIST supervised. Panel **B**: Models MarkerMap joint, Concrete VAE, Global-Gumbel VAE, RankCorr, Scanpy overestimate variance, Scanpy Wilcoxon with tie correction, COSG, and PERSIST unsupervised. Note that both panels use the same y-axis for direct comparison.

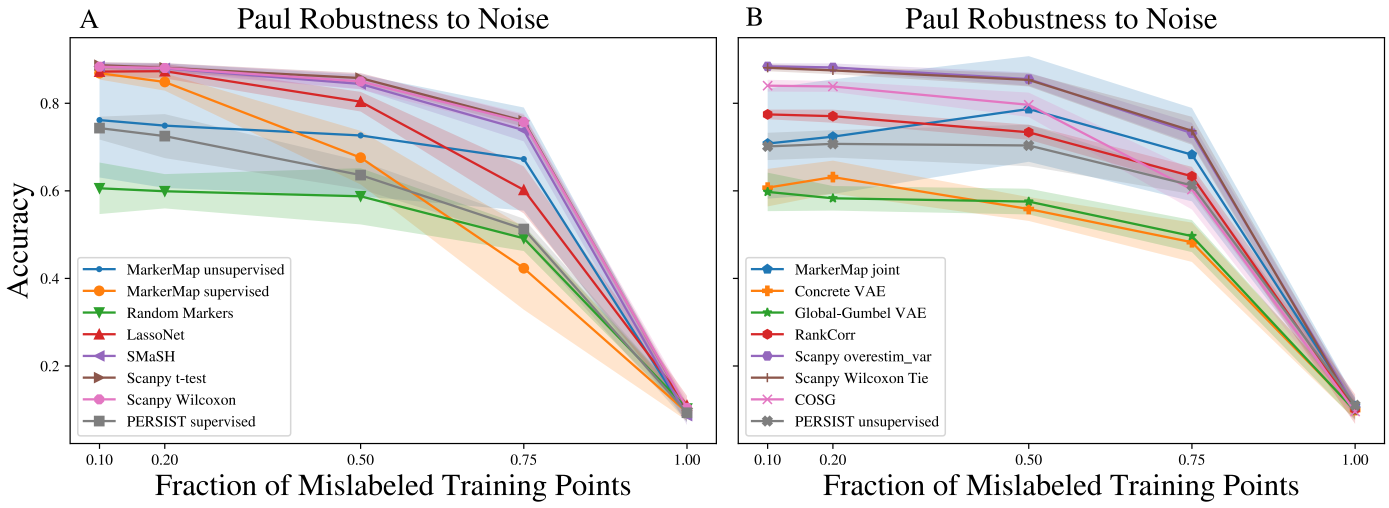

Supplementary Figure 9: Accuracy benchmark over label error using a Random Forest classifier for the Paul data set, averaged over 10 runs. Panel **A**: Models MarkerMap unsupervised, MarkerMap supervised, Random Markers, LassoNet, SMaSH, Scanpy t-test, Scanpy Wilcoxon, and PERSIST supervised. Panel **B**: Models MarkerMap joint, Concrete VAE, Global-Gumbel VAE, RankCorr, Scanpy overestimate variance, Scanpy Wilcoxon with tie correction, COSG, and PERSIST unsupervised. Note that both panels use the same y-axis for direct comparison.

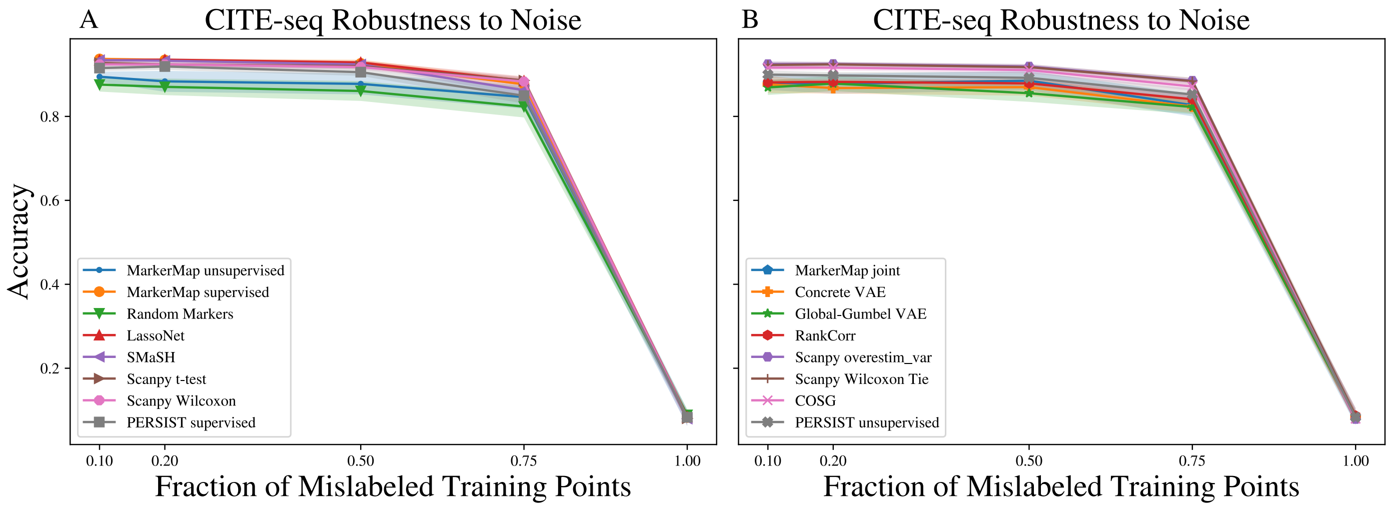

Supplementary Figure 10: Accuracy benchmark over label error using a Random Forest classifier for the CITE-seq data set, averaged over 10 runs. Panel **A**: Models MarkerMap unsupervised, MarkerMap supervised, Random Markers, LassoNet, SMaSH, Scanpy t-test, Scanpy Wilcoxon, and PERSIST supervised. Panel **B**: Models MarkerMap joint, Concrete VAE, Global-Gumbel VAE, RankCorr, Scanpy overestimate variance, Scanpy Wilcoxon with tie correction, COSG, and PERSIST unsupervised. Note that both panels use the same y-axis for direct comparison.

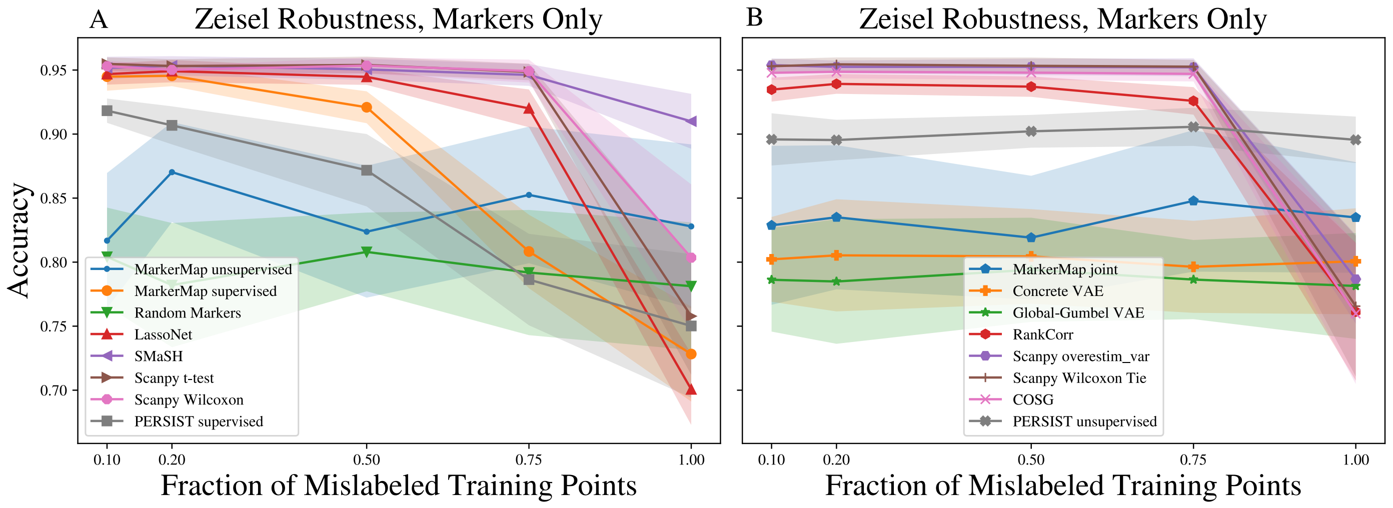

Supplementary Figure 11: Accuracy benchmark over label error while finding markers using a Random Forest classifier for the Zeisel data set, averaged over 10 runs. Panel **A**: Models MarkerMap unsupervised, MarkerMap supervised, Random Markers, LassoNet, SMaSH, Scanpy t-test, Scanpy Wilcoxon, and PERSIST supervised. Panel **B**: Models MarkerMap joint, Concrete VAE, Global-Gumbel VAE, RankCorr, Scanpy overestimate variance, Scanpy Wilcoxon with tie correction, COSG, and PERSIST unsupervised. Note that both panels use the same y-axis for direct comparison.

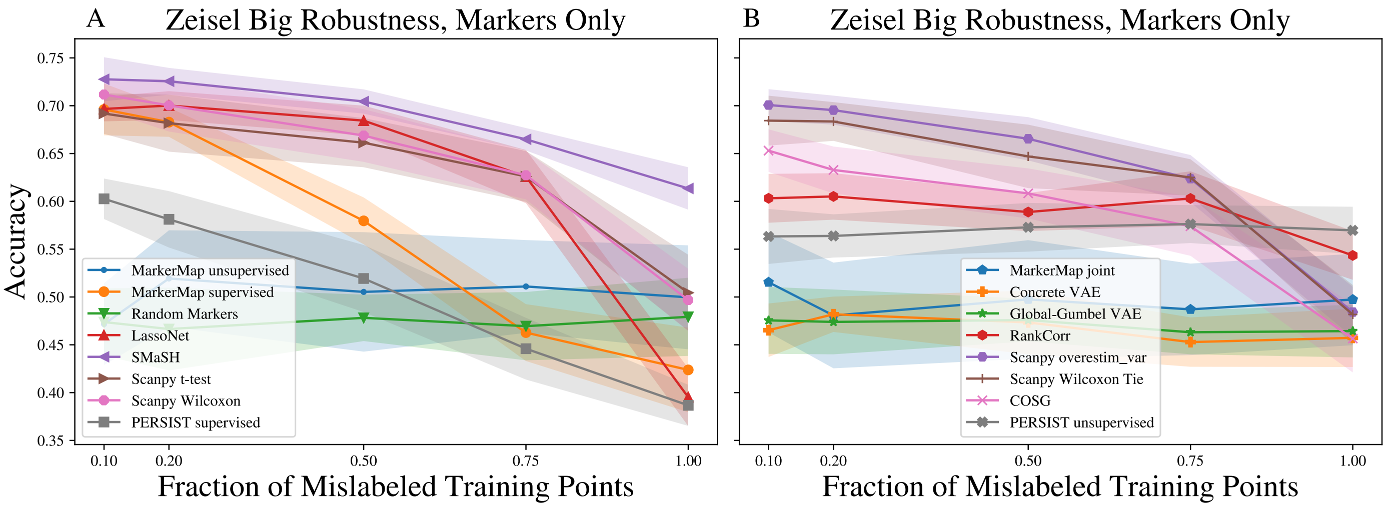

Supplementary Figure 12: Accuracy benchmark over label error while finding markers using a Random Forest classifier for the Zeisel Big data set, averaged over 10 runs. Panel **A**: Models MarkerMap unsupervised, MarkerMap supervised, Random Markers, LassoNet, SMaSH, Scanpy t-test, Scanpy Wilcoxon, and PERSIST supervised. Panel **B**: Models MarkerMap joint, Concrete VAE, Global-Gumbel VAE, RankCorr, Scanpy overestimate variance, Scanpy Wilcoxon with tie correction, COSG, and PERSIST unsupervised. Note that both panels use the same y-axis for direct comparison.

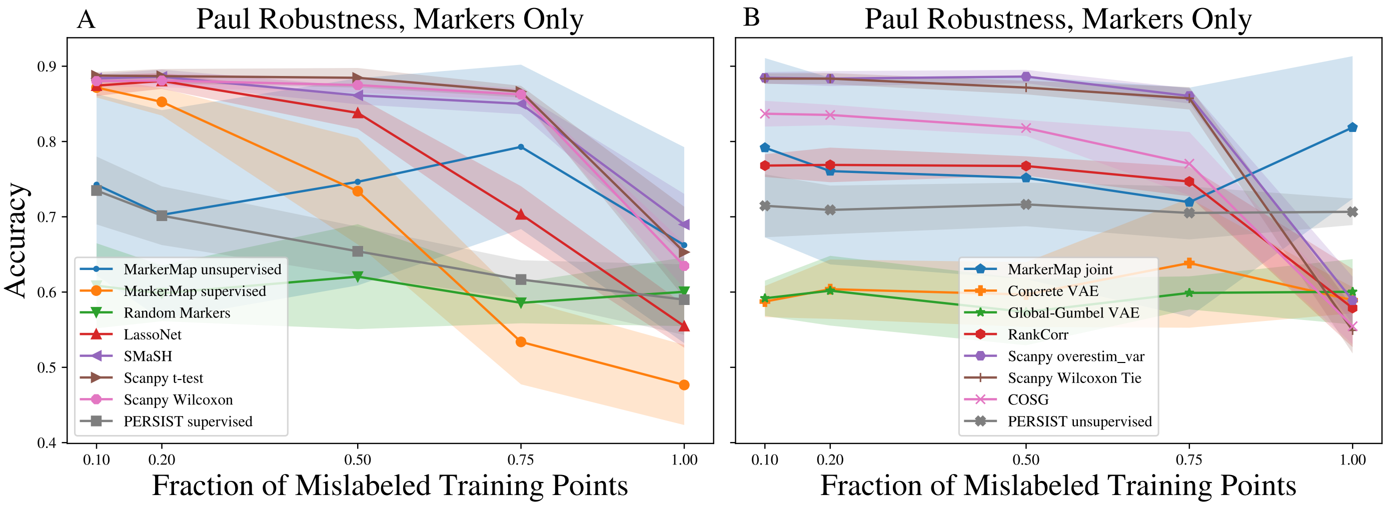

Supplementary Figure 13: Accuracy benchmark over label error while finding markers using a Random Forest classifier for the Paul data set, averaged over 10 runs. Panel **A**: Models MarkerMap unsupervised, MarkerMap supervised, Random Markers, LassoNet, SMaSH, Scanpy t-test, Scanpy Wilcoxon, and PERSIST supervised. Panel **B**: Models MarkerMap joint, Concrete VAE, Global-Gumbel VAE, RankCorr, Scanpy overestimate variance, Scanpy Wilcoxon with tie correction, COSG, and PERSIST unsupervised. Note that both panels use the same y-axis for direct comparison.

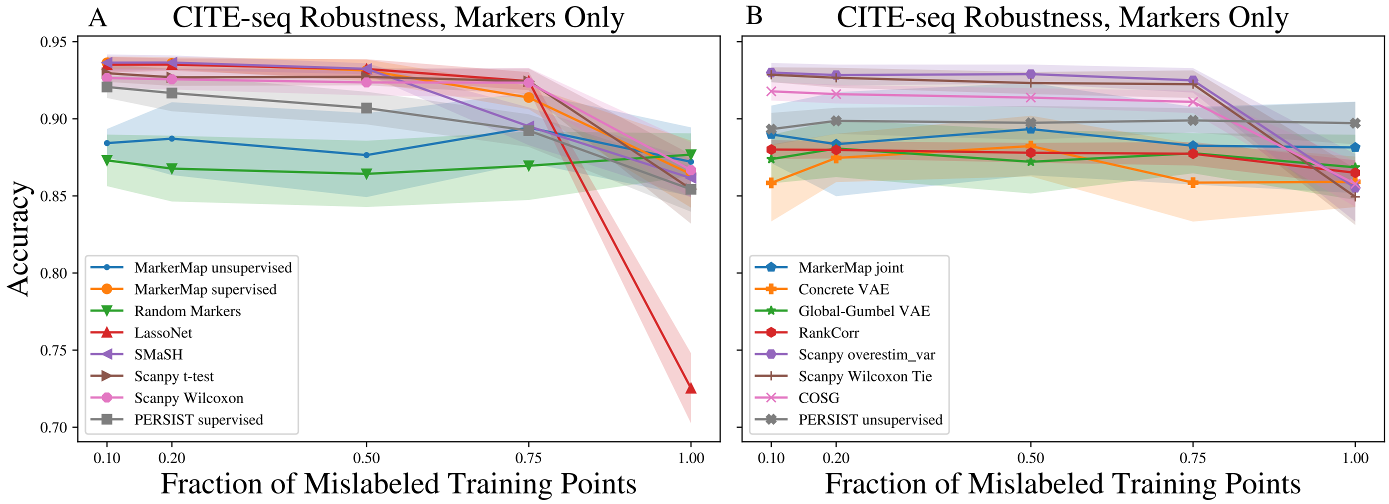

Supplementary Figure 14: Accuracy benchmark over label error while finding markers using a Random Forest classifier for the CITE-seq data set, averaged over 10 runs. Panel **A**: Models MarkerMap unsupervised, MarkerMap supervised, Random Markers, LassoNet, SMaSH, Scanpy t-test, Scanpy Wilcoxon, and PERSIST supervised. Panel **B**: Models MarkerMap joint, Concrete VAE, Global-Gumbel VAE, RankCorr, Scanpy overestimate variance, Scanpy Wilcoxon with tie correction, COSG, and PERSIST unsupervised. Note that both panels use the same y-axis for direct comparison.
